# Psychological well-being in Hungarian students with specific learning disorders (SpLD)

**DOI:** 10.1192/j.eurpsy.2022.599

**Published:** 2022-09-01

**Authors:** R. Dudok, B. Pikó

**Affiliations:** 1 University of Szeged, Doctoral School Of Education, Szeged, Hungary; 2 University of Szeged, Department Of Behavioral Sciences, Szeged, Hungary

**Keywords:** specific learning disorders, Adolescents, psychological well-being, resilience

## Abstract

**Introduction:**

The specific learning disorders (SpLD) are neurology based development disorders which are characterized by significant difficulties in acquiring learning skills. SpLD are often accompanied by mental health problems because of emotional and behavioral consequences of the diagnosis. While the presence of SpLD can worsen psychological well-being among school aged children, resilience factors can neutralize these harmful effects.

**Objectives:**

The aim of this research is to identify protective factors promoting development of psychologial well-being and mental health in adolescents with SpLD compared to a control group of peers without this diagnosis.

**Methods:**

Hungarian primary school students (N = 174; M = 13.34 years, SD = 1.14) participated in the study aged between 11 to 15 years. Of these, 23.6% students had a SpLD diagnosis. Participants completed a paper pencil-based questionnaire that included: EPOCH Psychological Well-being Questionnaires of Adolescent, WHO Well-being Index, Satisfaction With Life Scale, Multidimensional Perceived Social Support Scale, The Connor-Davidson Resilience Scale, and Self-regulation Scale.

**Results:**

Psychological well-being in both groups shows positive correlation (p<0.01) with general well-being, satisfaction with life, social support, self-esteem and self-control. In the SpLD group, the engagement and connectedness subscales do not relate to the general well-being and life satisfaction, in contrast with the control group in which these are associated with both. Self-control shows stronger correlation with general well-being and with engagement and connectedness subscales as compared to the control group (p<0.01).

**Conclusions:**

There are differences in contributing factors of general psychological well-being in adolescents with SpLD and those without this diagnosis.

**Disclosure:**

No significant relationships.

